# On the Use of the Matico

**Published:** 1846-07

**Authors:** W. S. W. Ruschenberger

**Affiliations:** Surgeon U. S. N.


*On the Use of the Matico.* By W. S. W. Ruschenberger, M. D., Surgeon U. S. N. Communicated by Professor Dunglison.

*U. S. Naval Hospital,*
New York, May 27th, 1846.

*My Dear Sir,*—Since I forwarded to you some notes on matico about a year since, I have used it frequently, and I have heard of its use by some of my friends. As a remedy for gonorrhœa, I do not think it is equal to cubebs. I made an experiment w h it to test its virtues as a styptic. In enlarging a burrowing bubo, I divided the arteria ad cutem abdominis, which bled freely. I directed that an attempt should be made to arrest the hem rrhage by lint and pressure. After a trial of ten minutes, which totally failed, I directed moistened matico leaves to be applied. The assistant reported in a few minutes that the matico exerted no influence, and proposed to secure the bleeding vessel by a ligature. I now visited the patient, who had lost six or eight ounces of blood, and was still bleeding. After coarsely powdering some matico leaves in the palm of my hand, I formed the mass into a paste with cold water; I then removed the clot, through which the arterial blood formed a passage of the size of a crow-quill; the blood flowed per saltam, forming a jet of at least three-fourths of an inch high. The paste was applied lightly with the fingers, and filled the wound. The surrounding skin was immediately sponged clean; the hemorrhage ceased instantly, and not a single drop of blood flowed afterwards. No pressure was used, or dressing applied.

In the first application the entire matico leaf had been simply dipped in water and then applied. It failed, as already stated.

A cupper and leecher in Brooklyn informs me, that he finds the matico, applied as I have described above, to instantly arrest the bleeding of leech-bites in children, which, before he obtained matico, frequently caused him great anxiety. He expressed himself as perfectly satisfied, that there cannot be any difficulty in arresting hemorrhage from leech-bites, where matico can be obtained.

I exhibited dram doses of finely powdered matico in a case of profuse menorrhagia, repeated every two hours. The flow ceased after the third dose. The powder was simply mixed in about two ounces of water. The patient experienced no unpleasant effects from its use.

Those who are desirous of trying the virtues of matico, may obtain the genuine article of Mr. Frederick Brown, Philadelphia.

Dr. J. H. Scrivener, in a letter dated Lima, April 30, 1845, says that matico grows abundantly along the sides of the mountains of Monobamba and Huanuco in the Department of Junin. There are three species distinguished by the color of their stems, which are red, brown and white. The red species is considered superior to the others, and is brought to Lima in large quantities by Indians, and sold to the druggists. When the Flora Peruana was published, the medicinal properties of the plant were unknown; their discovery is attributed to a soldier who was wounded on the 9th December, 1824, at the battle of Ayacucho.

The plant is very generally used in Lima, and along the coast in cases of hemorrhage, and all kind of ulcers. The leaves are well pounded and then applied to the wound; the vessels contract and the hemorrhage at once ceases. An infusion of the plant is used as a wash for ulcers; and after washing, a small quantity of the powder is applied. It does not appear that matico has ever been employed in Lima as an internal remedy.

There is sufficient evidence in favor of matico to render its properties and medical qualities worthy of careful examination; I say careful examination, because we may be deceived by the results of a few observations and experiments, and be lead to believe what is far from truth. We are too apt to arrive at conclusions without sufficient data; too apt to forget the proverb, "One swallow don't make summer," and hence come many of those facts which are known as *false.*

It is possible that all the medicinal qualities of the matico may be found to exist in its essential oil, which is heavier than a saturated solution of common salt. I am about causing some trials to be made with the article, which I have procured by the ordinary method of distillation.

I am, very truly, and respectfully, your friend,

W. S. W. Ruschenberger.

Dr. Robley Dunglison.

---

To the Editor of the Medical Examiner:

Below I send you a copy of a letter recently received from my friend Dr. Bell, of Waterbury, Ct., in reply to a letter which I addressed to him in regard to his paper on the "Use of Quinine in Typhoid Fever," published in the May number of the *Examiner*. It sheds further light upon a very interesting question in the therapeutics of a grave form of disease, and I have no doubt that its publication will be gratifying to the numerous readers of your ably conducted Journal.

I am, with great respect, your obedient servant,

Geo. L. Upshur.

*Waterbury*, (*Ct.*) May 15th, 1846.

*Dear Doctor:* It is with much pleasure that I answer your communication which came to hand on Saturday last. You ask,

"do you use the term *typhoid* as it is employed by most of the French writers, who make it a synonyme for *dothinenterite;* or simply as most American and English physicians use it, to signify a *low form* of fever?" Although the phrase *typhoid fever* is sanctioned by some of the best writers on that disease, both in France and this country, the name is not sufficiently current to be entirely free from misapplication, and dothinenterite is certainly much more definite and a better term.

I use the term *typhoid* with the same signification as it is used by Louis, Chomel, Andral and others of France, and by Jackson, Bartlett and Gerhard of this country—a *distinct disease,* made evident by the morbid alteration in the glands of Peyer, enlargement of the corresponding mesenteric glands, and frequently enlargement and alteration of the spleen; the common symptoms of which are chills, with or without shivering, and followed by heat and pespiration, usually partial; or hot, dry skin; great prostration; pain in the head, back, and limbs; diarrhœa, with pain in the abdomen; or costiveness; loss of appetite; great thirst; delirium; unrefreshing sleep; difficulty of attention; dizziness; drowsiness; flushed face, with eyes bright and injected, and eyelids weighed down; tinnitus aurium; hacking cough, and a panting snuffing respiration; frequent pulse, sometimes very tense and quick; tongue at first white or yellow; spleen can usually be felt. About the seventh day a majority of patients have an eruption principally confined to the abdomen and chest, composed of a few red papular elevations or pimples, which vanish upon pressure and immediately reappear. One week later there is a different eruption, *sudamina,* about the clavicles, axilla, epigastrium and inguinal regions; epistaxis is common at any stage of the disease, and other hemorrhages are not unfrequent; the tongue grows darker, swollen, glossy at the edges, parched, often cracked, and bleeds, is protruded with difficulty, and there is an inability to retract it except very slowly and carefully; thick sordes collect on the lips and teeth; abdomen becomes tumid and resonant from meteorism, and is often extremely tender, especially in the right iliac region; countenance more suffused, red or purplish, sometimes bloated; brow contracted; sunken, vacant and wild expression; subsultus tendinum, and picking at the bed-clothes and imaginary objects; sliding down in bed; diarrhœa; urine and dejections offensive; involuntary discharges; retention of urine; convulsive movements, or permanent coma; pulse 150 or upwards per minute; facies Hippocratica; sudden development of peritonitis, indicating perforation of the intestine, which is necessarily fatal.

But it is never to be expected that the whole of these symptoms will be found in one case, though it is *possible* that they all may occur at once. As to that of the "rose coloured eruption," which you are not alone in considering as a pathognomonic sign of typhoid fever, I believe it to be often wanting in the genuine form of the disease, but I have never seen a case in which it was absent *at the onset or just preceding* tympanitis and resonance of the abdomen with great tenderness, *and during that condition.* This I would have mentioned in my paper had I thought my observation extensive enough to justify such a conclusion. As far as the evidence of my cases go, however, I am induced to think that tympanitis and resonance of the abdomen with extreme pain, rarely occur without being preceded by, or simultaneous with, the rose coloured eruption; and although the eruption may occur without such an affection of the abdomen, it usually exists at the same time and in the same case.

I carefully looked for the eruption in every case published in my paper, but it was absent, nor did any of them have serious affection of the intestines, both of which I think were prevented by the use of quinine. You will find in "Andral's Clinique," Diseases of the Abdomen, case 70, that on account of the extreme debility of the patient on the eleventh day of a typhoid fever, he commenced the use of infusion of Peruvian bark, during the exhibition of which all the bad symptoms disappeared, amongst which were the petechiæ. From an error committed in diet, the eruption reappeared, but it seemed to exercise but little influence over the patient *during the exhibition of the bark.* Now, if such was the effect of the infusion of Peruvian bark, was it not by virtue of the quinine it contained? And if so small a portion acted so beneficially, now that the advantages of giving it in large doses are so well known, is it too much to assume, that the rose coloured eruption, and *that which it indicates*, viz. *serious affection of the intestines*, may be prevented in typhoid fever by a timely and liberal use of the sulphate of quinine?

I desire to test the effects of this remedy still further, before I shall feel entirely satisfied to answer the above interrogatories in the affirmative. If succeeding cases, however, are followed by the same results as those I have published, opportunity being afforded to commence the exhibition of quinine at the beginning of the eruption without the development of any more serious symptoms, I shall then, without hesitation, say, that in typhoid fever, the rose coloured eruption and its usual sequence may be prevented by the proper use of the sulphate of quinine.

I have thus answered, at some length, your questions, and as the same are liable to arise in the minds of others who may have read my article, you are not only at liberty, but it will be my pleasure, if you deem it of sufficient importance, to send this letter for publication in the next number of the *Examiner*.

Very respectfully, your friend,

A. N. Bell.

To Dr. G. L. Upshur, Norfolk, Va.

